# Estrogen-Related Receptor Influences the Hemolymph Glucose Content by Regulating Midgut Trehalase Gene Expression in the Last Instar Larvae of *Bombyx mori*

**DOI:** 10.3390/ijms22094343

**Published:** 2021-04-21

**Authors:** Guanwang Shen, Jinxin Wu, Ying Lin, Xiaoting Hua, Qingyou Xia, Ping Zhao

**Affiliations:** 1 Biological Science Research Center, Southwest University, Chongqing 400716, China; gwshen@swu.edu.cn (G.S.); wjx651268650@163.com (J.W.); ylin197266@163.com (Y.L.); huaxiaotingswu@126.com (X.H.); xiaqy@swu.edu.cn (Q.X.); 2 State Key Laboratory of Silkworm Genome Biology, Southwest University, Chongqing 400716, China

**Keywords:** estrogen-related receptor, trehalase, hemolymph glucose, transcriptional regulation, silkworm

## Abstract

The expression of trehalase in the midgut of insects plays an important role in glucose supply to the hemolymph. Energy metabolism is usually regulated by the estrogen-related receptor (ERR). A decrease in ATP levels is caused by the ERR hindering glycolysis. However, the relationship between trehalose accumulation and ERR expression is still unclear. Here, we found that silkworm ERR (BmERR) is concentrated and BmERR expression is strongly correlated with trehalase in the midgut during the last instar silkworm larval stage. We cloned the promoter of the trehalase from *Bombyx mori* (BmTreh) and found that the ERR bound directly to the core response elements of the promoter. Cell level interference and the overexpression of ERR can reduce or enhance BmTreh transcription and promoter activity. Overexpressed transgenic BmERR can significantly increase the expression of BmTreh in the midgut of the last instar silkworm larvae, thereby hydrolyzing trehalose into glucose and releasing it into the hemolymph. Additionally, increased hemolymph glucose content reduces silkworm pupa weight but does not affect silk protein production from the silk gland. Our results suggest a novel function for BmERR through its involvement in BmTreh regulation and expand the understanding of ERR functions in insect trehalose metabolism.

## 1. Introduction

Estrogen-related receptor (ERR) was first characterized as an orphan nuclear receptor (NR) belonging to the NR3 superfamily and has no natural ligand [1]. Giguere et al. obtained the cDNA of ERR from human cDNA libraries by utilizing the DNA-binding domain of the estrogen receptor (*ER*) gene as a probe using low-stringency hybridization techniques [2]. ERR is widely identified in vertebrates and exists in many isotypes; there are three paralogous genes in mammals, *ERRα*, *ERRβ*, and *ERRγ* [2,3], while there are five isotypes in zebrafish [4]. Compared to vertebrates, only one type of *ERR* was cloned in most invertebrates [5,6,7,8]. Although our understanding of the evolutionary history of the *ERR* gene is still incomplete, it is generally believed that ERRs are derived from a common ancestral morphology in both invertebrates and vertebrates. *ERR* redundancy occurred several times during the evolutionary transition from invertebrates to vertebrates [5,9].

ERRs in vertebrates play a key role in the regulation of energy metabolism: transcription factors and associated co-regulators modulate the energy metabolism pathway synergistically [10]. Previous studies have shown that some transcription factors involved in the NR family play crucial roles in this process; moreover, they transform hormonal, nutritional, and metabolite signals into specific gene expression networks to supply energy to life activities [11]. ERRs are very sensitive to associated co-regulators that are crucial for the control of energy homeostasis, and these co-regulatory proteins can be used as “protein ligands” for ERRs [10,12,13,14]. Additionally, as a transcription factor, ERR can directly bind to the ERR-binding element (ERRE) on the promoter of genes involved in energy homeostasis; this regulates expression and thus the glucose metabolism in cells and the oxidative phosphorylation in mitochondria [11,15,16].

In *Drosophila melanogaster*, *Bombyx mori,* and *Agrotis ipsilon*, only one *ERR* gene was characterized [17,18]. However, it plays an essential role in development and adaption. For example, this gene enhanced the response to sex pheromones in male adult *Agrotis ipsilon* [19], regulated the sex differentiation of *Hymenoptera: Formicidae* [20], promoted the proliferation of midterm embryonic cells in *Drosophila melanogaster*, and downregulated ERR in the testis of *Drosophila*, leading to abnormal testis development [21]. Furthermore, *ERR* can participate in the stress response to other abiotic factors in insects, such as *in Chironomus riparius* [22,23], *Apis cerana* [24], and *Diaphanosoma celebensis* [25]. ERR also responds to the ecdysone signal in *B. mori* [26], *Drosophila melanogaster* [27,28,29], *Polyrhachis vicina* [30], and *Orthoptera: Gryllidae* [19,31,32]. Recent studies show that glucose metabolism of *B. mori* [33], *Drosophila melanogaster* [27,34,35] and *Aphids* [36] are regulated by ERR. *ERR* knockout in *Drosophila melanogaster* significantly reduces the expression level of the the gene encoding the rate-limiting enzyme phosphofructokinase (*PFK*) in glycolysis [17]. Subsequent studies in silkworm confirmed that ERR regulates *PFK* expression by binding to the ERRE element on the promoter of the *PFK* gene [33].

Interestingly, in *ERR*-knockout *Drosophila*, the process of glycolysis was blocked, ATP synthesis was reduced, and trehalose was accumulated [17]. Trehalase (Treh) hydrolyzes trehalose into glucose in insects. As described before, the regulation of glucose utilization (promoting glycolysis) by ERR is an almost indisputable fact in mammals, *Drosophila melanogaster*, and *B. mori*. In this study, we used *B. mori* as the object of our research to focus on whether ERR could regulate the expression of *Treh* to promote the production of glucose in insects.

## 2. Results

### 2.1. Analysis of the Expression Pattern of ERR and Treh in Silkworms

**Figure 1:**
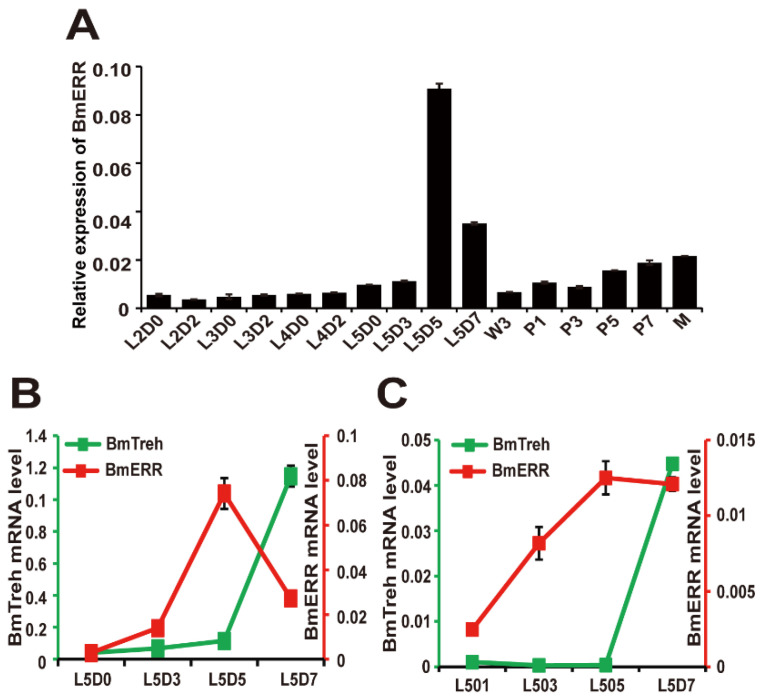
The expression profile of *BmERR* and *BmTreh* at different development stages of *B. mori*. (**A**) The expression profile of *BmERR* at different development stages; (**B**) The expression profile of *BmERR* and *BmTreh* in the midgut of the last instar *B. mori*; (**C**) The expression profile of *BmERR* and *BmTreh* in the silk gland of the last instar *B. mori*. L2/3/4/5D0 indicates the first/second/third/fourth instar molting larvae; L2/3/4/D2 indicates the second day of the second/third/fourth instar larvae; L5D/3/5/7 indicates the third/fifth/seventh day of fifth instar larvae; W3: day 3 of wandering; P1, P3, P5, and P7: days 1, 3, 5, and 7 of pupation, respectively; M: moths.

To investigate the possible function of BmERR, we first detected the transcription profile of BmERR over the entire life cycle of silkworm using quantitative real-time polymerase chain reaction (qRT-PCR). BmERR was mainly expressed during the last instar in silkworm larval stage, which begins at day 3 (L5D3) and peaks at day 5 (L5D5) during the silk protein synthesis stage (Figure 1A).

To determine whether BmERR influences the hemolymph glucose content by regulating Treh expression, we analysed BmERR and silkworm Treh (BmTreh) expression profiles in the midgut during the last instar. qRT-PCR results revealed that the expression of BmERR reached a peak at L5D5. Correspondingly, BmTreh expression reached a peak at L5D7 (Figure 1B). At this stage, silk glands are needed to absorb the great amount of glucose from the hemolymph to synthesize the silk protein provided energy. Glucose in the hemolymph of the last instar silkworm is mainly released from the midgut through the hydrolysis of trehalose into glucose by Treh. The continuous expression of BmERR during the silk protein synthesis period was also detected in the silk glands. However, the Treh gene in the silk glands was almost not expressed during this period. This indicates that the main function of ERR in the silk glands during this period is not to regulate the expression of BmTreh (Figure 1B).

In this study, we mainly focused on the regulation of ERR on subsequent Treh expression. As such, we aimed to determine whether BmERR plays a role in regulating the hemolymph glucose content of last instar silkworms by inducing the expression of BmTreh.

### 2.2. BmERR Regulated Treh Expression

**Figure 2:**
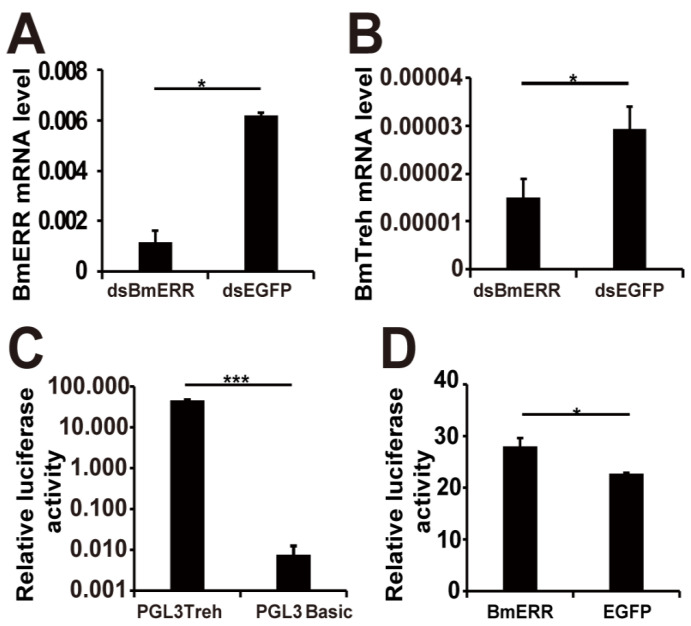
The expression of BmTreh is upregulated by BmERR in BmE cells. (**A**,**B**) BmTreh was downregulated by the RNAi of BmERR. qRT-PCR analysis of BmERR and BmTreh mRNA in dsBmERR treated cells; (**C**) Relative luciferase activity of the BmTreh promoter in BmE cells. (**D**) BmERR promote the luciferase activity driven by the promoter of BmTreh. The data represent means ± standard deviations (*n* = 3). The significance of the differences between data sets was calculated using two-tailed Student’s *t*-tests; * *p* < 0.05. *** *p* < 0.001.

To analyze whether BmERR was involved in the transcriptional regulation of BmTreh, an RNA interference (RNAi) assay for BmERR was conducted. RNAi assays were performed to detect the expression of BmERR in embryonic B. mori (BmE) cells. The results showed that when BmERR expression was decreased (Figure 2A), the expression of endogenous BmTreh was also decreased when compared to the control (Figure 2B).

The upstream 2000-base pair (bp) DNA fragment from the transcription start site of BmTreh was PCR-amplified by using the silkworm genomic DNA as the template. A pGL3-Basic plasmid containing a BmTreh Promoter (BmTreh P) and the luciferase gene was constructed. The constructs designated as pGL3-Treh were transfected into BmE cells, and then, promoter activity was assessed by measuring luciferase activity. As shown in Figure 2C, these cells were more capable of expressing luciferase compared to the control plasmid pGL3-Basic. BmE cells were then cotransfected with BmERR and BmTreh P Luc, after which the BmTreh P activity was increased (Figure 2D). These results indicate that BmERR might activate the expression of BmTreh.

**Figure 3:**
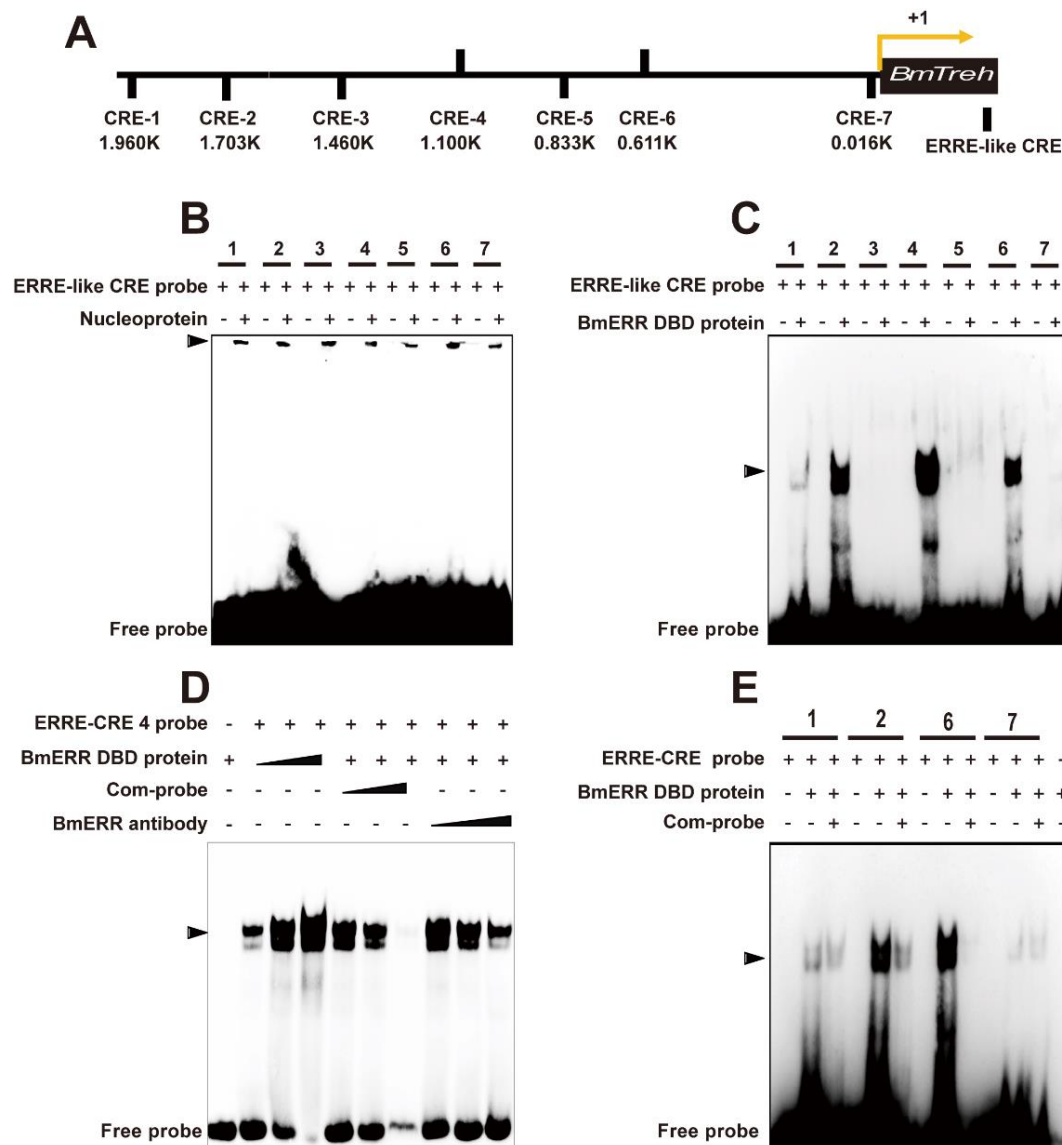
Identification of ERR core response elements (CREs) in the promoter of BmTreh in vitro. (**A**) Analysis of ERR CREs in the promoter of BmTreh; (**B**) The nucleoprotein extracted from the midgut at the last instar stage bind with the ERRE like CRE probe; (**C**) Prokaryotic ERR DNA-binding domain (DBD) protein bind with ERRE like CRE probe 1, 2, 4, 6and 7. (**D**) ERRE-like 4 specifically binds to BmERR DBD; (**E**). ERRE-like 1/2/6 specifically binds to BmERR DBD.

Given that BmTreh is regulated by BmERR, to determine how BmERR regulates BmTreh expression, we analyzed the 2000-bp sequence and identified ERREs. We predicted seven ERR core response elements (CREs) in the 2.0-kb region upstream of BmTreh P (Supplementary Figure S1), based on the conserved ERRE sequences using JASPAR (http://jaspar.genereg.net/, accessed on 13 June 2020), which were designated as ERRE-like CREs (Figure 3A). Incubation of a biotin-labeled ERRE-like probe with 5LD7 midgut nuclear extracts caused a noticeable band shift in the electrophoretic mobility shift assay (EMSA) (Figure 3B). To confirm which of the predicted seven ERRE motifs of the BmTreh P binds to the BmERR, we incubated biotin-labeled ERRE-like probes with recombinant BmERR DNA-binding domain (DBD) proteins. An EMSA showed a band shift that increased with ERRE-like CRE probes 1/2/4/6 (Figure 3C). We then randomly selected ERRE-like CRE 4 for further verification. When the ERRE-like CRE 4 probe was incubated with the BmERR DBD protein, there was a marked increase in the band shift with increase in BmERR concentration. This band narrowed markedly when an anti-BmERR antibody or competitive probe was added (Figure 3D). Moreover, ERRE-like CRE 1/2/6 probes bound to the BmERR protein and could be inhibited by competitive probes (Figure 3E).

**Figure 4:**
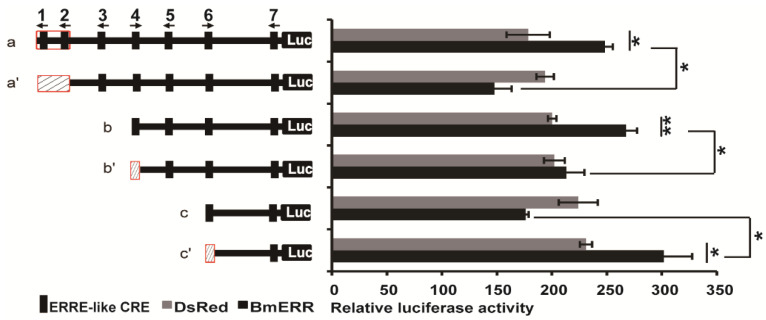
BmERR increased the activity of BmTreh promoter through core response elements—ERRE-like CRE 1,2,4 and 6 in embryonic silkworm cells. The significant difference between data sets was calculated using two-tailed Student’s *t*-tests; * *p* < 0.05. ** *p* < 0.01, NS: No significant difference.

To analyze whether ERR increased the activity of the BmTreh P via ERRE-like, we shortened the promoter to two lengths of its original size, with or without the 1/2,4,6 probes, in order to construct the cell transfection vector. The vectors were then cotransfected with the psl1180-Hr3-A4 BmERR Simian vacuolating virus 40 (SV40) overexpression vector into BmE cells. After BmERR was overexpressed, and the activities of the promoter with ERR CREs 1/2,4 were significantly higher than those without these elements (Figure 4: a vs. a’, b vs. b’). These results demonstrated that BmERR enhanced the activity of the BmTreh P through ERR CREs 1/2,4.

Furthermore, we found that after BmERR was overexpressed the luciferase activity decreased significantly when the ERR CREs 6 motif of BmTreh P was existed (Figure 4: c vs. c’). EMSA was preliminary confirmed that CRE 6 can directly bind to the BmERR protein. These results may suggest that CRE 6 site in the BmTreh P besides have the ability to bind BmERR, may are involved in other transcription factor’s (OTF) regulation of BmTreh. For example, OTF probably inhibits BmTreh P expression by competitively binding to the CRE 6 motif in the BmTreh P promoter, which can be proven, and further study is needed.

### 2.3. Overexpression of BmERR in Transgenic Species Increases the Expression of BmTreh and Hemolymph Glucose Content

**Figure 5:**
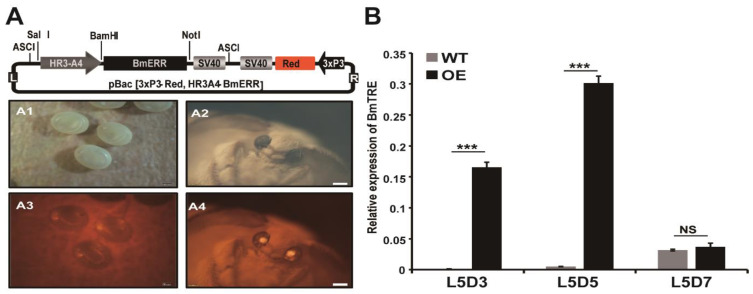
Transgenic overexpression of BmERR. (**A**) Schematic diagram of the BmERR transgenic overexpression vector; pBacR and pBacL are the right and left arms of the transposon, respectively; 3xp3 EGFP simian vacuolating virus 40 is the transgene marker; Hr3 A4 is the promoter. A1–4 Screening of positive transgenic *Bombyx mori* under a fluorescence microscope; (A1/2) white light image of transgenic *B. mori* eggs/moth; (A3/4) fluorescence image of transgenic *B. mori* eggs/moth (**B**) The expression of *BmTreh* in the midgut of BmERR transgenic lines. *** *p* < 0.001, NS: No significant difference.

To analyze the role of BmERR on the regulation of BmTreh as well as on the effects on hemolymph glucose content in silkworms, a piggyBac-based transgenic plasmid pBac- Hr3-A4 BmERR SV40 was constructed, containing Hr3-A4, BmERR, and a 240-bp 3′-end region of the SV40 poly A signal. Additionally, red fluorescent protein was utilized as the selecting marker by using an expression cassette driven by the 3xp3 promoter (Figure 5A). After overexpression of BmERR, the last silkworm instar midgut endogenous BmTreh expression was significantly increased (Figure 5B).

**Figure 6:**
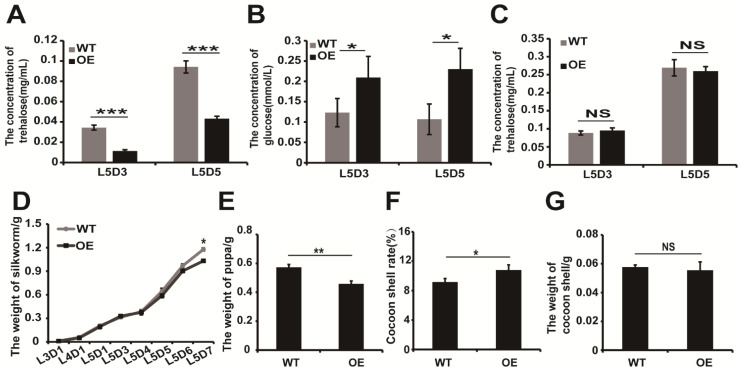
The effect of transgenic overexpression of silkworm estrogen-related receptor on the development of the last instar silkworm larvae. (**A**) Detection of trehalose content in the midgut of the last instar silkworm; (**B**,**C**) Detection of glucose/trehalose content in the hemolymph of the last instar silkworm; (**D**). Body weight during the feeding stage of the silkworm larvae. (**E**–**G**) Pupa weight/Cocoon shell ratios/Cocoon shell weight of silkworms. The data represent the mean ± standard deviations (*n* = 30). The significant difference between data sets was calculated using two-tailed Student’s *t*-tests; * *p* < 0.05; ** *p* < 0.01. *** *p* < 0.001.

Subsequently, we detected the content of trehalose on L5D3 and L5D5 in transgenic silkworm larvae. The trehalose content was significantly lower in transgenic silkworms compared to the wild-type silkworm midguts at the same time point (Figure 6A). However, the glucose content in the hemolymph increased significantly in the transgenic silkworms when compared to the wild-type silkworms at the same time point (Figure 6B). Additionally, we found that trehalose content did not significantly change in the hemolymph (Figure 6C). These results suggest that the overexpression of BmERR and the induction of midgut BmTreh transcription increased the hemolymph glucose content of the last instar silkworm.

Additionally, we examined the relationship between the hemolymph glucose content and the development of silkworms. We compared the body weight of last instar transgenic silkworm larvae with wild-types and found the larval body weight significantly decreased on the fifth day for the last instar transgenic silkworm (Figure 6D), whereas the pupa weight decreased (Figure 6E). This leads us to conclude that an increase in the cocoon shell ratio of transgenic silkworms was higher than that in wild-type silkworms (Figure 6F). However, cocoon shell weight displayed no significant difference compared to the wild-type silkworm (Figure 6G).

This study suggests the the role of BmERR in the silkworm midgut in promoting BmTreh expression and the subsequent hydrolysis of trehalose into glucose, which enter the hemolymph, increasing the glucose contentin the hemolymph. Moreover, excessive decomposition of trehalose in the last instar silkworm reduces the weight of the silkworm pupa and ultimately increases the cocoon shell ratio of the silkworm. However, this does not affect the silk protein production of the silk gland.

## 3. Discussion

The silkworm belongs to the family Lepidoptera: Bombycidae, it is an insect of complete metamorphosis. There are four main stages of its life: egg, larva, pupa, and moth. The larval stage lasts about 25–30 days and includes five instars. After completing four molts, the larvae develop into pupae. Because of its unique silk-producing characteristics, it has become an important economic insect. *Bombyx mori* has 28 pairs of chromosomes, and its genome contains almost 450 million bp. This demonstrates its abundant genetic polymorphism. *Bombyx mori* has many advantages in life science research due to its moderate size and ease of dissection, enabling many tissues and organs, such as midgut, fat body, silk gland, and hemolymph, to be easily obtained and analyzed [37]. With the completion of the *B. mori* Genome Project [38,39] and the establishment of the *B. mori* Genome and Protein Databases, it has gradually become a valuable research model and can be used instead of *Drosophila* [40,41,42].

The silk glands explosively grow in the last instar of silkworm larvae and account for 40% to 45% of the larval body weight, becoming the largest organ in the silkworm body by the end of silk production. A large amount of energy is produced via glucose hydrolysis and is consumed to produce synthetic silk proteins in the fifth instar. Therefore, having enough energy for the development of silk glands and synthesis of silk proteins is essential. The synthesis of silk protein requires hundreds of proteins and more than 30 RNAs. Enzymes related to glucose decomposition and energy supply, such as ATP synthase and glyceraldehyde 3 phosphate dehydrogenase, are highly abundant in the silk glands of the last instar silkworm larva [43,44].

Silkworm glucose is mainly hydrolyzed from trehalose by BmTreh [45,46,47]. BmTreh activity is the highest in the midgut; as such, the glucose produced by BmTreh in the midgut can then re-enter the hemolymph and be absorbed and utilized by other tissues [48,49,50]. In this study, we found that the expression levels of BmTreh in silk glands were low, less than one twentieth of that in the midgut. This may suggest that the energy for silk protein synthesis was mainly provided by absorbing glucose released into the hemolymph that was originally from the midgut. BmERR binds with the ERREs in the BmTreh P to upregulate the expression of BmTreh. The period expression profile demonstrated that BmERR was mainly expressed in the midgut during the fifth instar of silkworms. Thus, the glucose required for silk protein synthesis in the last instar silkworm larva silk gland may have been absorbed from the hemolymph.

The last instar silkworm larvae consume more than 80% of the amount of food than that for the total instar larval period; this accumulated nutrition is mainly used to synthesize silk protein for cocooning and pupation. Compared to wild-type silkworms, the upregulation of *Treh* in the midgut causes significantly elevated glucose levels in the hemolymph of transgenic lines, while the weight of larvae in both the wandering and pupa stages decreased. This may suggest that the expression of *Treh* is induced by BmERR in the midgut; thus, the enzyme breaks down trehalose into glucose to provide energy for silk production. Furthermore, the amount of trehalose in the hemolymph did not change. Thus, it is supposed that the increase in glucose content in the hemolymph is due to the metabolism of both trehalose and glycogen; trehalose was released from the fat cells and into the hemolymph to keep the trehalose levels stable. Thus, the nutrients used for pupation after cocooning were prematurely consumed.

In addition, overexpression of ERR in this study had no significant effect on silk protein production. Moreover, the increase in the cocoon layer ratio was mainly caused by a decrease in pupa weight. The elevation of the glucose level in the hemolymph improved the efficiency of silk protein synthesis in silk glands. However, more work is needed to confirm this by combining our results with the dynamic detection of silk protein content during the fifth larval stage.

The development of silk glands at the last larval stage is characterized by rapid protein synthesis, which requires the efficient conversion of nutrients into protein. We found that the expression of ERR is upregulated in the fifth larval stage during silk protein synthesis. However, the relationship between sustained expression of BmERR in silk glands and the energy required for silk protein synthesis has not been fully elucidated. In the future, studies should explore the relationship between the expression of ERR, glucose decomposition, and ATP generation during silk protein synthesis, as this may provide new insights.

## 4. Materials and Methods

### 4.1. Animals

The silkworm strain D9L was provided by the Silkworm Gene Bank (Southwest University, Chongqing, China). The silkworm was reared with fresh mulberry leaves at a temperature of 25 °C ± 1 °C and treated with 12 h of light and 12 h of dark every day.

### 4.2. RNA/DNA Extraction and cDNA Synthesis

The whole body and midgut was collected for every growth period of the silkworm. These were then stored at −80 °C after freezing in liquid nitrogen. The tissues were ground after freezing with liquid nitrogen. Thereafter, the total RNA was extracted with Trizol extraction kit (Invitrogen, Carlsbad, CA, USA) and stored at −80 °C. The first-strand cDNA of tissues was synthesized with the M-MLV reverse transcriptase (Promega, Madison, WI, USA). Genomic DNA was extracted from the whole silkworm using the Tissue DNA kit (OMEGA, Norcross, GA, USA) according to the manufacturer’s instructions.

### 4.3. qRT-PCR

Using the NovoStar SYBR SuperMix Plus (Novoprotein, Beijing, China) and the ABI StepOne v2.1 Sequence Detection System (Applied Biosystems), a qRT-PCR assay was performed to evaluate the mRNA levels of BmERR and BmTreh were performed as previously described [26]. The primers used for the PCR are listed in Supplementary Table S1.

### 4.4. Bioinformatics Analysis and Vector Construction

According to the position of predicted ERRE-like motif on the BmTreh P, different primers were designed to acquire different lengths of promoter fragments from the silkworm genomic DNA. These constructs were then inserted into the firefly luciferase reporter vector pGL3-Basic (stored in our lab) between the SacI and XhoI restriction sites to form different recombinant vectors (primers are shown in Supplementary Table S1). The cellular overexpression vector (psl1180-HR3-A4-BmERR-Ser1PA and psl1180-HR3-A4-DsRed- Ser1PA) were stored in our laboratory.

### 4.5. Cell Transfection, Luciferase Assay, and Double-Stranded RNA Interference Assay

The luminescence reporter assays were performed according to the manufacturer’s instructions using the Dual-Luciferase Reporter Assay System (Promega, Madison, WI, USA). The BmE cell line (stored in our laboratory) was used in this study. They were cultured at 27 °C in Grace’s insect medium (Gibco, Grand Island, NE, USA) supplemented with 10% fetal bovine serum (FBS; Gibco, Grand Island, NE, USA). The cells were placed in a 24-well cell culture plate 12 h prior to transfection. Thereafter, a mixture of 1 μg recombinant plasmid, 0.1 μg internal control plasmid, and 3 μL of Lipofectamine 2000 (Invitrogen, Carlsbad, CA, USA) in the insect medium without FBS was introduced into each well. After 8 h, the transfection mixture was replaced with fresh 500 μL insect medium containing 10% FBS. The fragments of BmERR and BmEGFP containing the bacteriophage T7 promoter sequence were obtained through PCR (primer sequences are shown in Supplementary Table S1) and cloned into the pMD19-T simple vector. The double-stranded RNA was generated using the T7 RiboMAX Express RNAi System (Promega, Madison, WI, USA).

### 4.6. EMSA

According to the nucleotide sequence of predicted ERRE-like motifs on the BmTreh P, the 3′-end biotin-labeled probes (shown in Supplementary Table S1) were synthesized. To evaluate interactions between regulatory elements and prokaryote-expressed BmERR DBD protein (Refence 26/33, stored in our laboratory), an EMSA was performed using a Chemiluminescent EMSA Kit (Beyotime, Beijing, China) according to the manufacturer’s instructions. After incubating at 25 °C for 20 min, reaction mixtures were loaded into 5% native polyacrylamide gels and electrophoresis was then conducted in Tris-borate–EDTA buffer (45 mM Tris-borate and 1 mM EDTA, pH 8.3). The proteins were transferred to a nylon membrane (Thermo, Waltham, MA, USA). The detection of the bound horseradish peroxidase-conjugated antibodies was performed by enhanced chemiluminescence and imaging using the Clinx ChemiScope 3400 Mini system (Science Instruments, Shanghai, China).

### 4.7. Construction of Transgenic Silkworms

In order to construct the transgenic vector piggybac (3×p3-DsRed-SV40, HR3-A4-BmERR-SV40), the HR3-A4-BmERR-SV40 fragment was obtained by PCR and attached to the transgenic backbone vector using homologous recombination. The gene amplification conditions were as follows: 98 °C for 3 min, 35 cycles of 98 °C for 10 s, 56 °C for 20 s, and 72 °C for 40 s, followed by a final extension period of 72 °C for 10 min. The primers used are listed in Supplementary Table S1. PrimeSTAR^®®^ Max DNA Polymerase (TAKARA, Kyoto, Japan) was used for the PCR. The homologous recombination was performed by using Ligation Free CloningKit (abm, Shanghai, China) according to the manufacturer’s instructions.The eggs of D9L laid within 6 h were microinjected with a mixture of recombinant transgenic plasmid (400 ng/μL) and helper plasmid (400 ng/μL). The injected eggs were incubated at 25 °C until they hatched and were then reared with a diet of fresh mulberry leaves. The G1 embryos and moths were screened for DsRed-positive expression under a fluorescent microscope (Olympus, Kyoto, Japan). Transgenic lines were screened and designated as OE-BmERR for the subsequent detection experiments.

### 4.8. Weight Statistics

The weights of wild-type D9L and OE-BmERR during different instars were measured. The weights of the cocoon, pupa, and cocoon shell were also calculated when the silkworms were in the third day of pupal period (*n* = 30).

### 4.9. Measurement of Trehalose and Glucose Levels

The hemolymph and midguts were obtained from six silkworms. The hemolymph was centrifuged at 1000× *g* for 10 min at 4 °C, and the supernatant was collected. The midguts were mixed and divided into 0.1 g/portion. The trehalose and glucose contents were determined using to the Trehalose Content Assay Kit (Solarbio, Beijing, China) and Glucose Content Assay Kit (LEAGENE, Beijing, China), respectively.

### 4.10. Statistical Analysis

Statistical values are shown as means ± SEM. Mean values were compared using the Student’s *t*-test with the following significance thresholds: * *p* < 0.05, ** *p* < 0.01, and *** *p* < 0.001. Statistical analyses were performed using Graph Pad Prism 6.0 (Graph Pad software, San Diego, CA, USA). Figures were assembled using Adobe Photoshop CS5 and Adobe Illustrator CS5.

## 5. Conclusions

BmERR is mainly expressed in the midgut of the last instar silkworm larva. BmERR binds to CREs in the BmTreh P, thus upregulating the expression of BmTreh as well as increasing the glucose content in the hemolymph of the last instar silkworm larvae. This method of regulation may be conserved in all insects. These results suggest a new function of BmERR in trehalose metabolism regulation, thus expanding the understanding of the ERR in insect development. All these results provide a new scientific basis for improving the understanding of the glucose metabolism network of ERR in insects. Other functions of the ERR may require further investigation in other insect species. An in-depth understanding of the mechanism of ERR in insects will promote research on the evolution of these species and the relationship between invertebrates and vertebrates.

## Data Availability

Not applicable.

## Supplementary Materials

The following are available online at https://www.mdpi.com/article/10.3390/ijms22094343/s1, Figure S1: Upstream region sequence of the BmTreh gene. Table S1: The primers and probes used for this study.
