# Supplementary figures and images for: Estrogen-Related Receptor Influences the Hemolymph Glucose Content by Regulating Midgut Trehalase Gene Expression in the Last Instar Larvae of Bombyx mori
Source: Int J Mol Sci. 2021 Apr 21;22(9):4343. doi: 10.3390/ijms22094343 (PMC8122577; doi:10.3390/ijms22094343)

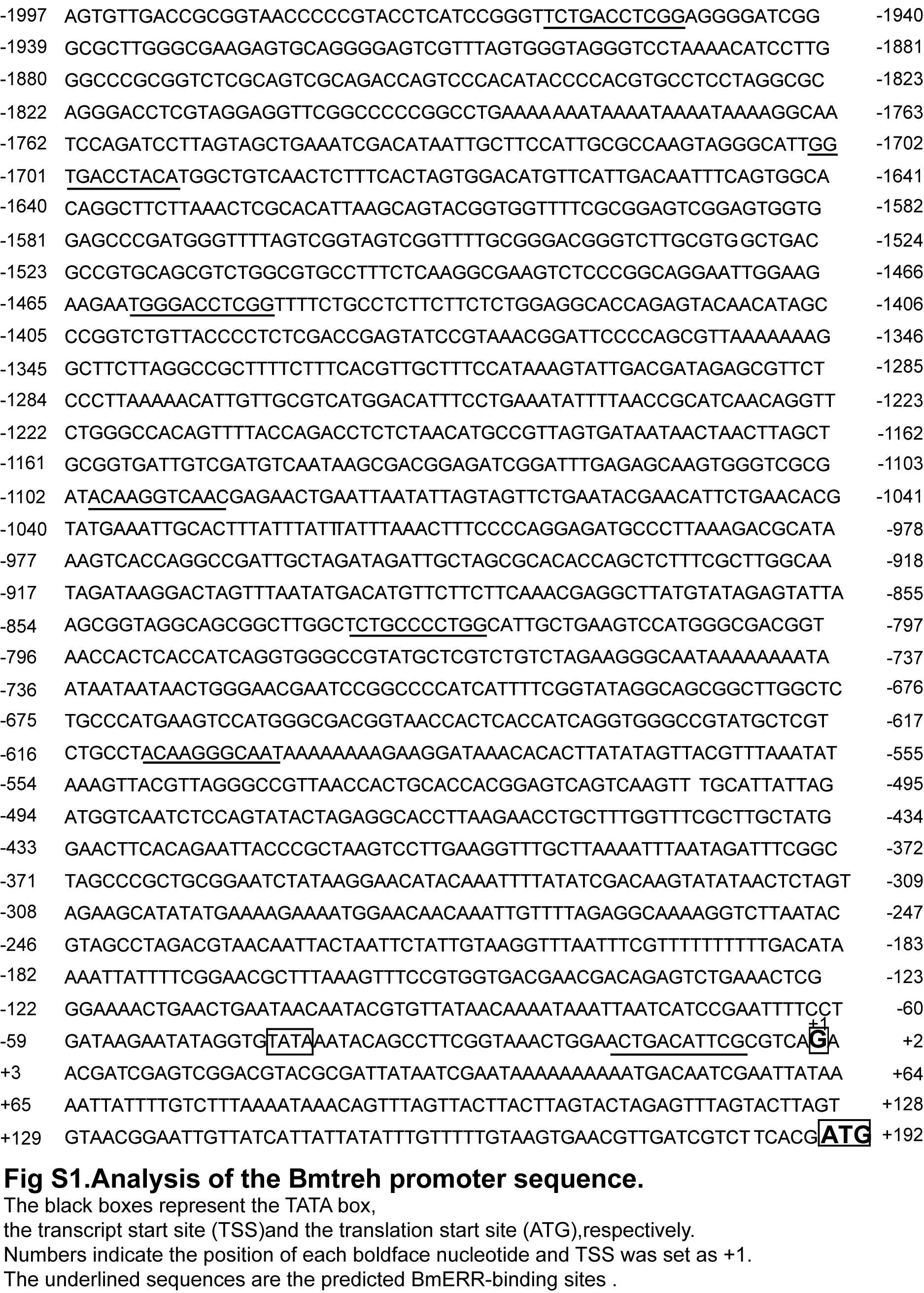

Supplement: Supplementary file 1 [file ijms-22-04343-s001.zip › Fig S1.tif]
